# Applying the balanced scorecard to local public health performance measurement: deliberations and decisions

**DOI:** 10.1186/1471-2458-9-127

**Published:** 2009-05-08

**Authors:** Erica Weir, Nadine d'Entremont, Shelley Stalker, Karim Kurji, Victoria Robinson

**Affiliations:** 1 Public Health Branch, Community and Health Services Department, Regional Municipality of York, Newmarket, Canada; 2 Institute of Medical Science, University of Toronto, Toronto, Canada

## Abstract

**Background:**

All aspects of the heath care sector are being asked to account for their performance. This poses unique challenges for local public health units with their traditional focus on population health and their emphasis on disease prevention, health promotion and protection. Reliance on measures of health status provides an imprecise and partial picture of the performance of a health unit. In 2004 the provincial Institute for Clinical Evaluative Sciences based in Ontario, Canada introduced a public-health specific balanced scorecard framework. We present the conceptual deliberations and decisions undertaken by a health unit while adopting the framework.

**Discussion:**

Posing, pondering and answering key questions assisted in applying the framework and developing indicators. Questions such as: Who should be involved in developing performance indicators? What level of performance should be measured? Who is the primary intended audience? Where and how do we begin? What types of indicators should populate the health status and determinants quadrant? What types of indicators should populate the resources and services quadrant? What type of indicators should populate the community engagement quadrant? What types of indicators should populate the integration and responsiveness quadrants? Should we try to link the quadrants? What comparators do we use? How do we move from a baseline report card to a continuous quality improvement management tool?

**Summary:**

An inclusive, participatory process was chosen for defining and creating indicators to populate the four quadrants. Examples of indicators that populate the four quadrants of the scorecard are presented and key decisions are highlighted that facilitated the process.

## Background

All aspects of the healthcare sector are being asked to account for their performance and to demonstrate efficiency and effectiveness in providing services to their clients. This requirement poses unique challenges for local public health units, with their traditional focus on population health and their emphasis on disease prevention, health promotion and health protection. Multiple factors determine public health outcomes [1], such as socio-economic factors, lifestyle, gender and genetics, yet only a few of these factors fall directly under the local health unit's programmatic responsibility and influence (Table 1). In Ontario, Canada, health units are mandated to provide a limited range of programs [2] – and are resourced accordingly. Consequently the overall health status of the residents within a health unit [3] presents only a partial and imprecise picture of the performance of the health unit.

**Table 1:** 1997 Ontario mandatory health programs and services guidelines

**Standard**	**Goal**
Equal Access	To ensure that all Ontarians have access to public health programs.
Health Hazard Investigation	To prevent or reduce adverse health outcomes resulting from exposure to health hazards as defined in the *Health Protection and Promotion Act *and including biological, physical, and chemical agents, natural or manmade.
Program Planning and Evaluation	To ensure that local programs address the health needs of the community, with cost-effective, efficient, evidence-based approaches.
Chronic Disease Prevention	To reduce the premature mortality and morbidity from preventable chronic diseases.
Early Detection of Cancer	To reduce mortality from breast cancer and cervical cancer by increasing early detection.
Injury Prevention Including Substance Abuse Prevention	To reduce disability, morbidity and mortality caused by motorized vehicles, bicycle crashes, alcohol and other substances, falls in the elderly and to prevent drowning in specific recreational water facilities.
Sexual Health	To promote healthy sexuality.
Reproductive Health	To support healthy pregnancies.
Child Health	To promote the health of children and youth.
Control of Infectious Diseases	To reduce the incidence of infectious diseases of public health importance.
Food Safety	To improve the health of the population by reducing the incidence of food-borne illness.
Infection Control	To reduce transmission of infectious diseases.
Rabies Control	To prevent the occurrence of rabies in humans.
Safe Water	To reduce the incidence of water-borne illness in the population.
Sexually Transmitted Diseases (STDs) Including HIV/AIDS	To reduce the incidence of and complications from all sexually transmitted diseases (STDs) including HIV/AIDS.
Tuberculosis (TB) Control	To reduce the incidence of tuberculosis (TB).
Vaccine Preventable Diseases	To reduce the incidence of vaccine preventable diseases.

In the past few years a growing number of healthcare provider organizations have adopted the balanced scorecard (BSC) framework to develop a more comprehensive set of performance indicators. The BSC is a management tool, originally applied to businesses in the private sector, developed by Kaplan and Norton in 1992 [4]. Its creators describe it as "a multidimensional framework for describing, implementing and managing strategy at all levels of an enterprise by linking objectives, initiatives and measures to an organization's strategy" [4]. Their tool broadened the traditional notion held by private sector companies that performance is indicated by financial measures solely, by integrating financial measures with other key performance indicators linked to three additional areas: customer preferences, internal business processes and organization growth, learning and development. A BSC includes performance measures in all four quadrants.

About a decade after Kaplan and Norton developed the BSC, a number of healthcare organizations in various healthcare settings throughout North America and abroad started to adapt and implement the BSC framework for their organizations. In Ontario, for example, over the past few years Cancer Care Ontario [5], the Ontario Hospital Association [6] and the University Health Network [7] have all adopted the BSC as their performance management tool. The four original quadrants were slightly modified to better reflect performance of publicly funded healthcare organizations rather than for-profit private businesses. The financial quadrant contains indicators of efficiency and asset utilization. The customer preferences quadrant contains measures of quality care and seamless service. The business process quadrant contains measure of continuous quality improvement and integrated service design and the learning and growth quadrant contains measures of human capital and strategic competencies.

**Figure 1:**
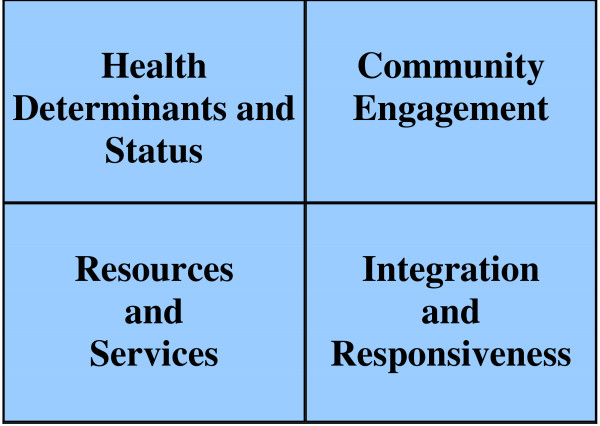
**Four quadrants of the ICES balanced scorecard for public health**.

In 2004 the Institute for Clinical Evaluative Science (ICES), based in Ontario, Canada, released a report, "Developing a BSC for Public Health" [8] that introduced a public health specific BSC framework for performance measurement. Public health's focus on prevention and health promotion, often for entire populations, distinguishes it from many other areas of healthcare that are more patient and treatment focused. The four quadrants were further adapted to include not only traditional measures of performance such as health status, but also measures relating to the structures and processes within the local public health unit (Figure 1).

The integrity of the original Kaplan and Norton framework is evident in the parallels between the original and proposed ICES quadrants. Traditionally businesses have relied fairly exclusively on the financial quadrant and the magnitude of profit generated as a measure of performance and public health units have relied on measures of the health status of residents within the health unit's jurisdiction. Whereas businesses focus on the level of customer satisfaction as a measure of performance, local public health units may also report on their levels of community engagement, since their customers and clients are populations and partners within the health unit's jurisdictions. Both businesses and health units may focus on their internal processes to demonstrate continuous quality improvements. In public health units this is may be measured by service delivery levels and efficiencies in resource distribution. And whereas corporations focus on continuous learning and demonstration of innovation to an ever changing market, public health practice must keep abreast of evolving population health science and evidence and demonstrate rapid responsiveness to emerging diseases and shifting political priorities.

In 2006, the Ontario Capacity Review Committee was appointed to lead a review of the organization and capacity of Ontario's local public heath units in the aftermath of the Severe Acute Respiratory Syndrome (SARS) outbreak. The committee recommended that health units produce annual reports for their funders and general public based on the ICES BSC [9]. In 2007, the Regional Municipality of York (See additional file 1 for profile of York Region) decided to join the group of early adopters and apply the BSC framework to the development of public health performance in the local health unit. This paper describes some of the initial key questions and conceptual challenges faced and decisions made in order to adopt a BSC framework.

## Discussion

### Who should be involved in developing the performance indicators?

The BSC provides a framework for reporting about performance. It also has the potential to be a management tool that aligns strategic direction with internal processes and instils understanding of and engagement in continuous quality improvement. To embrace these latter intentions an inclusive, participatory approach to indicator selection and development was chosen. A BSC panel was struck, comprised of three interested staff members, (i.e. a Director, manager and front-line staff person) from each of the 5 Divisions within the public health branch (i.e. Child and Family Health, Health Protection, Healthy Lifestyles, Infectious Disease Control, Dental and Nutrition) along with representatives from supporting services (e.g. community development, business services, epidemiology, emergency response). Borrowing from an earlier precedent, [10] the panel participated in a facilitated Delphi exercise to discuss and develop indicators to populate the four quadrants.

### Who is the primary intended audience?

The BSC potentially has multiple audiences: the local Board of Health and the provincial Ministry who share fiscal responsibility, the staff who plan and deliver programs, the general public who receive public health services and senior administration who set strategic direction. Our primary intent was to demonstrate accountability to our elected Board of Health and to provide them with information to inform decisions about mandate and resource allocation. As a result our audience for this initial BSC was the local Board of Health. The final version of the BSC was widely disseminated to staff, senior administrators, the provincial Ministry of Health and Long Term Care, federal and provincial agencies of public health and peer health units.

### What level of performance should be measured?

Some of the initial discussion among the panel members focused on the level of performance measurement. A main question that arose was 'Should the scorecard report performance at the overall health unit or at the division -specific (i.e. programmatic) level?' The original intent of the project was to develop an efficient scorecard for the health unit by populating a "dashboard" with a few key indicators aligned either with strategic priorities or particular themes, such as health inequity. However, we soon discovered that the participatory process involving representatives from all divisions and from three levels of staff invited and supported the development of a much more comprehensive and larger number of indicators that mostly described performance at the program-specific level. It became evident that the various program areas were not familiar with the activities in other areas and different programs began to compete for representation of their activities in the scorecard. Consequently, in order to build trust among different program areas and to garner sustained buy-in for the process and product, we decided to make the first scorecard comprehensive and include a large number of indicators, anticipating that over time and in future iterations the number and meaningfulness of indicators would be refined according to their relevance, utility, reliability and alignment with strategic priorities.

### How do we begin?

Over a period of two years, the team was able to operationalize the BSC, develop performance measures and raise awareness about continuous quality improvement among the health unit staff. The BSC implementation team consisted of an epidemiologist and a continuous quality improvement co-ordinator, who both dedicated 0.2 FTE over the course of the project. The office of the Medical Officer of Health championed the exercise. A consultant familiar with the BSC [10] was hired to initiate the process. Orientation sessions were held with the staff to educate them about the meaning of the BSC and about the health status of the residents within the catchment area for the health unit. The consultant led a series of four half-day facilitated exercises with the panel to rank and select indicators for the health determinants and status, community engagement and integration and responsiveness quadrants. To develop indicators about key activities, and the level of service activities and dedicated resources, the BSC team facilitated discussion with each program area. The panel and program areas submitted their selected indicators and provided text to describe their key activities. The result was a 75 page report containing 46 tables of indicators posted on the York Region website. [11].

### What types of indicators should populate the health status and determinants quadrant?

According to the ICES document [8], this quadrant contains measures traditionally found in community health status reports, such as trends in disease mortality/morbidity and health behaviours. The primary purpose is to identify the need for public health services. Ultimately this quadrant should contain three types of measures: determinants of population health, population health status measures and population health intervention impact measures. In Ontario, a lot of these data are routinely collected through provincial and federal surveys and made available to health units. Since the number of indicators is potentially very large, the panel was asked to select key indicators that described the burden of illness and the key demographic and social features that characterize York Region as unique. To assist the panel in choosing indicators for this quadrant the following guiding questions were posed: Whom does the York Region Public Health Branch serve? What are the health needs of the residents? Where does the burden of illness lie?

### What types of indicators should populate the resources and services quadrant?

According to the ICES document [8], the primary purpose of this quadrant is to report the amount of resources and services delivered in the health unit so that comparisons may be made across health units. The types of indicators that should be reported include indicators of financial resources in terms of per capita and total costs, the number of services delivered and the number of staff allocated to different services. To assist in indicator development for this quadrant the following guiding questions were posed: What are the key activities of the health unit? Who is the target population for these activities? Where is the bulk of our resources going?

### What types of indicators should populate the community engagement quadrant?

According to the ICES document [8], the views and involvement of the community who utilize local public health services and are impacted by public health policies need to be included as part of a public health performance report. Understanding the views of the population a program serves is a fundamental component of accountability and can improve the way services are delivered. Client satisfaction surveys are one of the traditional ways to invite input and feedback. Ideally community engagement should go well beyond client satisfactions surveys and encompass community and partner involvement in program planning, evaluation and service delivery (Table 2). Questions to guide the development of indicators for this quadrant included: How is the health unit engaging the community? How does the health unit ensure community input into public health planning and service delivery?

**Table 2:** Examples of indicators for the four quadrants

Quadrant	Indicator	Value
Health status and determinants	Teen pregnancy rate	49.8 per 1,000 young women aged 15–19 years
	Percentage of overweight or obese adults aged 18+	45.8%
Resources and services	Total number of investigations of institutional outbreaks per year	101
	Per capita spending for safe water program	$1.40
Community engagement	Proportion of current programs that ever consulted target population in needs assessment	47%
	Proportion of current programs that have completed a formal program evaluation	22%
Integration and responsiveness	Proportion of staff receiving emergency preparedness training in past year	100%
	Total number of peer reviewed journal publications, conference presentation and posters	57

### What types of indicators should populate the integration and responsiveness quadrant?

This fourth quadrant relates to the structural capacity of public health to keep it well integrated into the healthcare system as well as the capacity to continually transform services in response to evolving needs, issues and evidence (Table 2). This is achieved through the development of partnerships with local health service providers and community agencies. These partnerships have a mandate that impacts health determinants, through a commitment to research and academic pursuits, and through a corporate emphasis on continuing professional development and quality improvement. Questions to guide indicator development in this quadrant included: How are prevention, promotion and protection services integrated into the local healthcare system? How does the health unit identify and respond to emerging issues and evidence? How are continuing professional development and competency ensured?

### How do we collect the data?

Criteria for indicator development were established (See additional file 2 for criteria for indicator selection) and a data dictionary built to record a definition for each indicator. Responsibility for data collection and verification was distributed throughout the various program areas and involved the frontline staff who directly delivered the programs. Each program had a BSC champion, usually the same individual who participated in the Delphi panel, who worked with an epidemiologist to collect and verify the data.

### Should we try to link the quadrants?

There was a lot of initial deliberation about whether the BSC should attempt to link the quadrants to "tell a story". A hypothetical example would be exploring the relationship between the rate of motor vehicle collisions and high alcohol use in the health unit (health status and determinants quadrant). Currently the health unit dedicates 0.1 FTE to this issue through the substance abuse and injury prevention team (resources and services quadrant). There may be effective engagement between the health unit and the school board in promoting responsible drinking. The health unit may not have developed strategic alliances with the police (community engagement quadrant) and there is emerging evidence that reducing the legal alcohol limit for driving and promoting designated drivers programs is associated with a lower motor vehicle collision rate (integration and responsiveness quadrant). Based on this assessment, a decision to enhance resources in this program area may be made together with the establishment of a partnership with the police department to advocate for a change in legislation.

As this example illustrates, it is fairly easy to tie the quadrants together at the program level, but this is more difficult to accomplish at the overall health unit level. The above example does not touch on the numerous activities in the other mandated areas (Table 1). To attempt to weave stories around each activity for the purposes of reporting would result in information overload for the BSC report. Consequently our first BSC report was comprised of tables of indicators with little text to provide narration or interpretation. We decided to rely on the reader to interpret and draw conclusions based on the performance indicators, particularly when the health unit's performance was poor. However, program areas were encouraged to begin linking the quadrants as they moved forward with 2008 program planning and implementation on the basis of their reported 2007 performance.

### What comparators do we use?

Our initial report contains baseline measures that present a snapshot of the health unit's performance. Indicators are more meaningful when comparators are used. There are many options for comparators. One of the best options is to compare with oneself over time and look for trends in improvement. We intend to do this in future reports by comparing the baseline data with subsequent years. Other possible sources of comparison might be peer health units, as constructed by Statistics Canada [12], or provincial averages. Peer groups are better comparators than provincial averages since these health units share similar socio-economic characteristics that in part determine health status. However, since York Region is one of the first health units to develop indicators under the four quadrants suggested by ICES, comparable indicators from peer health units are not presently available. In order to encourage uptake of this framework among our peers, York Region invited members of neighbouring health units to participate as observers in the Delphi panel process.

### How do we move from a report card to a continuous quality improvement management tool?

The Board of Health received the BSC report at Committee and Council and requested a report back in six months explaining the action taken by the health unit in light of its performance. To assist with this, the BSC team facilitated focus groups with the various program areas and offered guidance questions such as: Which of these performance measures prompt you to change the way you do business? Each Division has been asked to identify three measures and to explain their impact on program planning and delivery. After reviewing the 46 tables, the indicators highlighted by program areas for response are those that most resonate with them. Through this selection process we will further identify common themes and use them to establish and inform strategic direction and future programmatic priorities. The BSC process is iterative and plans to refine and improve the selected indicators and our performance is ongoing.

### What are the lessons learned so far?

After completing our first BSC, we conducted a process evaluation with the staff who had been involved with performance measure development. In general the staff experienced the development of the scorecard as an informative and valuable exercise, but questioned the utility and relevance of some of the indicators as aids in program specific decision making. They recommended that a process be established to revisit and refine indicators and that more interpretation of indicators be provided in the text of the report. These finding are quite similar to the results reported by the Toronto-based University Health Network in the early phases of implementing their BSC [7]. Overall the University Health Network staff felt the University Health Network was moving in the right direction with its BSC but improvements were needed to make it more responsive and relevant to the University Health Network staff.

York Region Public Health Branch is in the process of producing our second report card based on the original indicators. As we revisit them, it is clear that some are not reliable or relevant and require refinement or removal. The program areas have been tasked with identifying variances from baseline and offering explanations, which will be provided in our second scorecard and presented to our governing Board.

## Summary

Tips for implementation of a BSC in a public health unit based on the BSC process experienced by the Public Health Branch of York Region:

• Involve management and front-line staff in the development of indicators to increase the likelihood of understanding, uptake and sustainability

• Start with a large number of indicators to reduce the chance of overlooking a set of indicators, and refine them over time and through iterations

• Target the initial scorecard at the governing board with fiscal responsibility since buy-in at the top level prompts future iterations and refinement of the process and product

• Consider using a Delphi exercise to generate discussion and consensus over indicators because it has been used successfully in the past and was demonstrably so in the Regional Municipality of York

• Indicators will become more informative over time as trends emerge and staff become more familiar with their relevance, particularly across divisions and programs

## Abbreviations

BSC: Balanced scorecard; ICES: Institute for clinical evaluative sciences.

## Competing interests

The authors declare they have no competing interests. The opinions stated are those of the authors and not of the Regional Municipality of York.

## Authors' contributions

EW, ND, SS, KK, VR conceived of the project and participated in the conceptualization of the quadrants and indicators and facilitation of the Delphi exercise. EW drafted this manuscript and ND, SS, KK and VR reviewed it and offered comments and their final approval.

## Author information

EW is a community medicine specialist and the Associate Medical Officer of Health for York Region. SS an epidemiologist, NdE is the co-ordinator of quality assurance and KK is the Medical Officer of Health for York Region. VR is a population health consultant who facilitated the Delphi exercise.

## Pre-publication history

The pre-publication history for this paper can be accessed here:



## Supplementary Material

Additional file 1 **Profile of the York Region Health Unit** Click here for file

Additional file 2 **The criteria for indicator selection** Click here for file
