# Supplementary material for: Applying the balanced scorecard to local public health performance measurement: deliberations and decisions
Source: BMC Public Health. 2009 May 8;9:127. doi: 10.1186/1471-2458-9-127 (PMC2684743; doi:10.1186/1471-2458-9-127)
Supplement: Additional file 1 — Profile of the York Region Health Unit [file 1471-2458-9-127-S1.doc]

York Region Profile

York Region is a large health unit in Ontario, Canada. It is composed of a population of a million people. It spans an area that extends from the northern border of the City of Toronto to the southern tip of Lake Simcoe. There are 9 municipalities in the Region. It is one of the 14 health units in Ontario where the board of health is aligned with a regional or municipal structure. The remaining 22 health units in Ontario have independent boards of health. York Region is one of the fastest growing health units in Ontario, with a population growth of 22.4% from 2001 to 2006. In 2006 immigrants composed 42.9% of the population compared to a provincial average of 28.3%. Overall the residents are relatively well off. The median after tax family income in 2006 was $70,000. However, about 10% of the population have incomes below Statistic Canada’s low-income cut-off (LICO). The population is aging, has a relatively higher birth rate than most other areas in Ontario and is experiencing the overweight/obesity epidemic that pervades at a level consistent with the provincial rate (circa 50%).
