# Supplementary material for: Applying the balanced scorecard to local public health performance measurement: deliberations and decisions
Source: BMC Public Health. 2009 May 8;9:127. doi: 10.1186/1471-2458-9-127 (PMC2684743; doi:10.1186/1471-2458-9-127)
Supplement: Additional file 2 — The criteria for indicator selection [file 1471-2458-9-127-S2.doc]

Criteria for indicator selection

Built on consensus

Based on a conceptual framework

Valid

Sensitive

Specific

Feasible

Reliable

Understandable

Timely

Comparable

Easy to collect

Flexible for use at different organizational levels
